# Molecular basis of domain‐specific angiotensin I‐converting enzyme inhibition by the antihypertensive drugs enalaprilat, ramiprilat, trandolaprilat, quinaprilat and perindoprilat

**DOI:** 10.1111/febs.70232

**Published:** 2025-08-18

**Authors:** Kyle S. Gregory, Vinasha Ramasamy, Edward D. Sturrock, K. Ravi Acharya

**Affiliations:** ^1^ Department of Life Sciences University of Bath UK; ^2^ Department of Integrative Biomedical Sciences, and Institute of Infectious Disease and Molecular Medicine University of Cape Town South Africa

**Keywords:** angiotensin I‐converting enzyme, antihypertensive drugs, domain selectivity, X‐ray crystallography, zinc metalloprotease

## Abstract

Angiotensin I‐converting enzyme (ACE) is a dipeptidyl carboxypeptidase with two homologous catalytic domains [N‐ and C‐domains (nACE and cACE)] that can cleave a range of substrates. cACE primarily cleaves the inactive decapeptide angiotensin I into the potent vasopressor angiotensin II, whereas nACE preferentially cleaves the antifibrotic tetrapeptide *N*‐acetyl‐seryl‐aspartyl‐lysyl‐proline (Ac‐SDKP). Several ACE inhibitors, which bind to both cACE and nACE active sites, are used clinically for the treatment of hypertension; however, serious side effects are seen in ~ 20–25% of patients due to nonselective inhibition. To improve ACE inhibitor side effect profiles, the design and development of selective inhibitors of cACE or nACE is desirable for the treatment of hypertension or fibrosis. The detailed molecular basis through which the clinically available ACE inhibitors bind and inhibit cACE and nACE was unknown. Thus, in this study, we have characterised the structural and kinetic basis for the interaction between cACE and nACE with enalaprilat, ramiprilat, trandolaprilat, quinaprilat and perindoprilat. The inhibitors display nanomolar inhibition of both domains, with moderate‐to‐low cACE‐selectivity. Trandolaprilat possesses the highest affinity for both nACE and cACE, whereas quinaprilat displayed the largest cACE‐selectivity. None of the binding modes of the inhibitors extend beyond the S1–S2′ subsites to make use of the unique nACE/cACE residues that have been shown to influence domain selectivity. These findings supplement our understanding of ACE inhibition by the clinically used ACE inhibitors, and this information should be useful in the future design of more domain‐selective inhibitors for the treatment of hypertension and cardiovascular diseases.

## Graphical abstract

Inhibition of angiotensin I‐converting enzyme is an effective strategy for the treatment of hypertension. However, the clinically available ACE inhibitors cause side effects due to nonselective inhibition of the two catalytic domains of ACE (nACE and cACE). Here, we present the kinetic and structural characterisation of nACE and cACE with the ACE inhibitors enalaprilat, ramiprilat, trandolaprilat, quinaprilat and perindoprilat to supplement our understanding of domain selectivity. Figure was produced using ccp4mg.

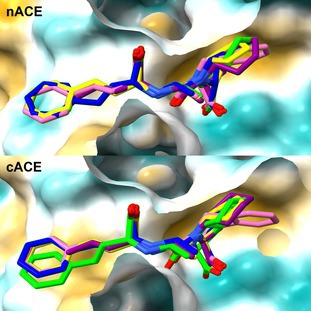

**Abbreviations**
ACE: angiotensin I‐converting enzyme
Ang I: angiotensin I
Ang II: angiotensin II
cACE: angiotensin I‐converting enzyme C‐domain
nACE: angiotensin I‐converting enzyme N‐domain
RAAS: renin–angiotensin–aldosterone system
sACE: somatic angiotensin I‐converting enzyme
tACE: testis angiotensin I‐converting enzyme

## Introduction

Angiotensin I‐converting enzyme (ACE, EC 3.4.15.1) is a zinc‐ and chloride‐dependent dipeptidyl carboxypeptidase that is involved in the regulation of blood pressure and electrolyte balance through the renin–angiotensin–aldosterone system (RAAS). Its main function is the cleavage of two peptides at the C terminus of the inactive polypeptide angiotensin I (Ang I), forming the potent vasopressor angiotensin II (Ang II) [1, 2, 3, 4]. Inhibition of ACE is therefore an effective strategy for the treatment of hypertension, and there are currently 17 ACE inhibitors in clinical use [5]. In addition to the hydrolysis of Ang I and other Ang peptides that make up the RAAS (Ang (1–9), Ang (1–7) and Ang (1–5)) [6, 7], ACE is able to cleave a wide range of substrates, including the anti‐inflammatory and antifibrotic tetrapeptide Ac‐SDKP [8], the vasodilatory peptide bradykinin [9], amyloid‐β [10, 11, 12], substance P [13], gonadotropin‐releasing hormone [14], neurotensin [15] and enkephalins [16]. This wide range of substrates highlights the role of ACE in many biological processes in addition to blood pressure regulation.

In somatic tissue, somatic ACE (sACE) exists as a heavily glycosylated type I transmembrane glycoprotein (1277 amino acids) with a large N‐terminal extracellular domain that is connected by a short linker to a transmembrane region, followed by a cytosolic tail [17]. The extracellular domain consists of two related catalytic domains, known as the N‐ and C‐domain (nACE and cACE, respectively), which share ~ 60% sequence identity and possess a conserved zinc‐binding motif (HExxH) that is essential for substrate hydrolysis [18]. In male germinal cell lines, an additional lower molecular weight isoform, testis ACE (tACE), is expressed, which is involved in the maturation of sperm cells [19]. The sequence of tACE is identical to that of cACE, except that tACE has an additional 36 residues at its N terminus that are heavily O‐glycosylated and possess an unknown function. Although nACE and cACE can hydrolyse the same substrates, cACE primarily processes Ang I [20], while nACE primarily cleaves Ac‐SDKP [21]. Therefore, cACE is the major contributor to blood pressure regulation, while nACE is involved in fibrosis. Both domains hydrolyse bradykinin equally [9]; thus, inhibition of nACE and cACE leads to the accumulation of bradykinin. The currently available ACE inhibitors are nonselective, inhibiting both nACE and cACE and have been shown to cause side effects in 20–25% of patients due to elevated bradykinin levels [9, 22]. Therefore, there is a need to design more selective ACE inhibitors that preferentially target either nACE or cACE for the treatment of inflammation, fibrosis, postmyocardial infarction and hypertension, which would be associated with fewer side effects. In addition to differences in substrate specificities, cACE was shown to have an increased dependency on chloride, which is an allosteric activator of ACE [23, 24, 25, 26, 27, 28], with the previously determined crystal structures of cACE and nACE containing two and one chloride ions, respectively.

One of the first ACE inhibitors, enalaprilat (Table 1), was designed in 1977 in the absence of an ACE crystal structure through structure‐based drug design, using insights obtained from the crystal structure of carboxypeptidase A [29, 30]. Subsequently, the crystal structure of cACE in complex with enalaprilat revealed zinc coordination through a carboxylate zinc‐binding group [24]. Enalaprilat consists of a tripeptide analogue (Phe‐Ala‐Pro) that extends into the S1 subsite via its phenyl group (P1) and occupies the S2' subsite via its proline moiety. Ramiprilat, trandolaprilat, quinaprilat and perindoprilat are also tripeptide analogues and are predicted to bind similar to enalaprilat; however, they vary by the number of hydrocarbons they possess at either the P1 or P2′ moieties. These inhibitors are administered as prodrugs (enalapril, ramipril, trandolapril, quinapril and perindopril), which contain an ethyl ester at the zinc‐coordinating carboxylate group, which must be metabolised into the active form [31]. In order to understand the molecular interactions of enalaprilat, ramiprilat, trandolaprilat, quinaprilat and perindoprilat with their binding sites, we have crystallised these inhibitors with nACE and cACE (excluding cACE:enalaprilat) and determined their 3D structures at high resolution using X‐ray crystallography. In addition, we determined the inhibitor constant (*K*_i_) and domain selectivity (*K*_i_(N)/*K*_i_(C)) of these inhibitors by fixed‐time fluorometric assays using the substrate Z‐Phe‐His‐Leu. These data will be useful in the future design of additional, more domain‐selective ACE inhibitors.

**Table 1:** Characterisation of nACE and cACE inhibition by enalaprilat, ramiprilat, trandolaprilat, quinaprilat, and perindoprilat.

Inhibitor	Structure (prodrug)	*K*_i_ (nm)		Selectivity^a^	Log*P* ^b^
		nACE	cACE		
Enalapril(at)	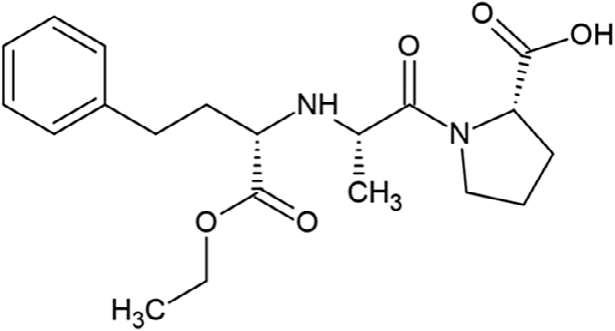	3.85 (±0.39)	1.06 (±0.15)	3.6	−0.7
Ramipril(at)	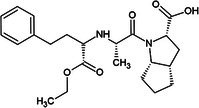	3.91 (±0.54)	0.21 (±0.04)	18.6	0.7
Trandolapril(at)	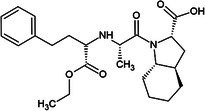	1.76 (±0.11)	0.15 (±0.01)	11.7	1.2
Quinapril(at)	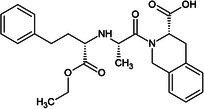	44.68 (±3.34)	1.28 (±0.32)	34.9	0.5
Perindopril(at)	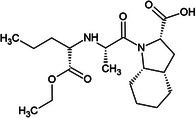	5.80 (±10.52)	0.83 (±0.15)	7.0	0.2

^a^ The cACE‐selectivity is calculated using: *K*_i_(nACE)/*K*_i_(cACE).

^b^ Log*P* values were calculated using xlogp3.3.0.

## Results and Discussion

### Inhibition kinetics and domain selectivity

**Fig. 1:**
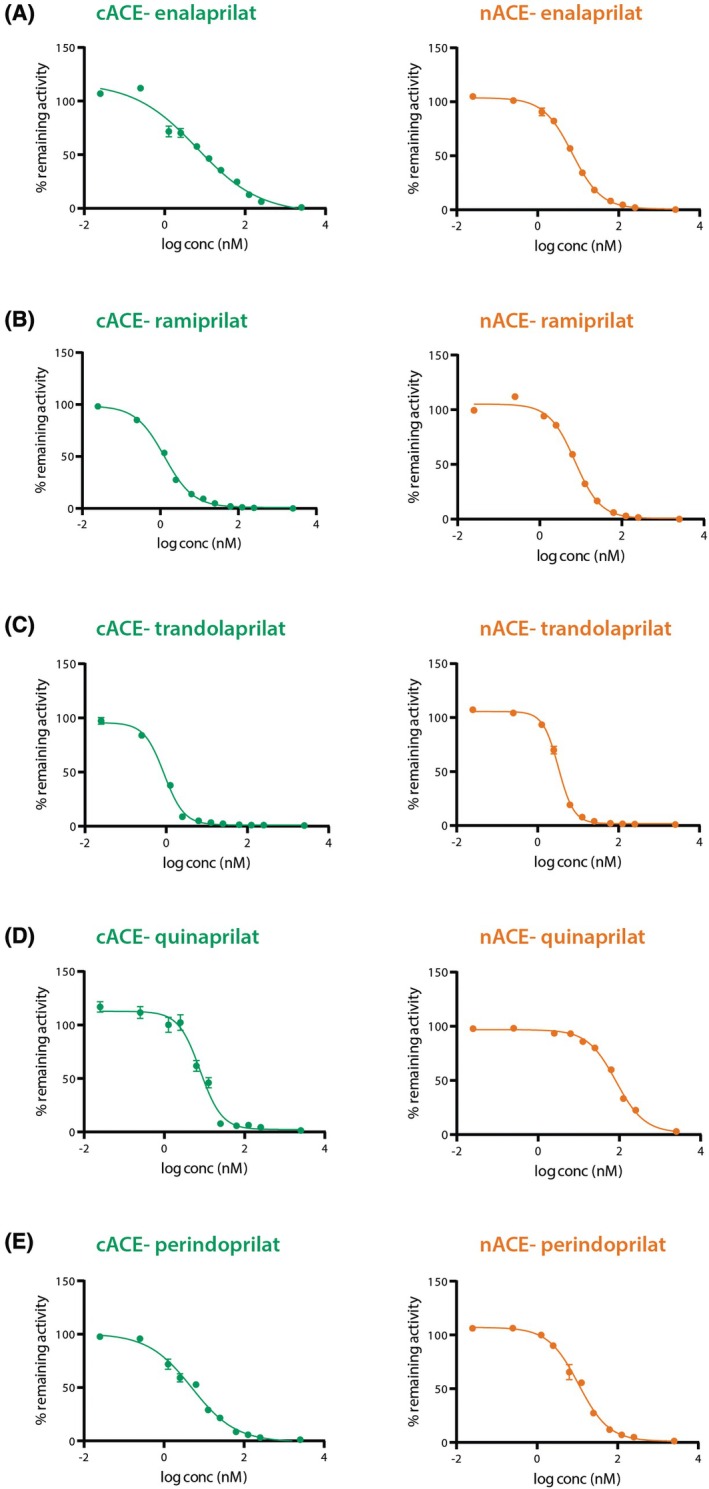
Inhibition curves for the soluble C domain (cACE) and N domain (nACE) of angiotensin I‐converting enzyme (ACE) with various ACE inhibitors. Representative half maximal inhibitory concentration (IC_50_) curves plotted for (A) enalaprilat, (B) ramiprilat, (C) trandolaprilat, (D) quinaprilat, and (E) perindoprilat. Assays (*n* = 3) were conducted using the peptide Z‐FHL in the presence of varying concentrations of the respective inhibitors.

To assess the strength of inhibition of nACE and cACE by enalaprilat, ramiprilat, trandolaprilat, quinaprilat and perindoprilat, kinetic analysis using a modified Z‐phenyl‐alanyl‐histidyl‐leucine (Z‐FHL) substrate was performed (Fig. 1); *K*_i_ values were calculated. To achieve maximal enzyme activation, we performed the assay in the presence of 300 mm NaCl as previously described [32]. The binding affinity (*K*_i_) and cACE‐selectivity (*K*_i_(N)/*K*_i_(C)) values are presented in Table 1. All the inhibitors tested in this study had nanomolar inhibition for nACE and cACE. Quinaprilat had the weakest inhibition, but the highest (albeit relatively poor) cACE‐selectivity. The minimal selectivity and similar potencies of the tested inhibitors is likely to contribute to the lack of clinically meaningful differences in antihypertensive efficacy previously reviewed by Heran *et al*. [33].

### Crystal structures of nACE:enalaprilat, nACE:ramiprilat, nACE:trandolaprilat, nACE:quinaprilat, nACE:perindoprilat, cACE:ramiprilat, cACE:trandolaprilat, cACE:quinaprilat, and cACE:perindoprilat complexes

Crystals of nACE:ramiprilat, nACE:quinaprilat, nACE:trandolaprilat, nACE:perindoprilat, cACE:ramiprilat, cACE:quinaprilat, cACE:trandolaprilat and cACE:perindoprilat were obtained through co‐crystallisation. Co‐crystallisation trials of nACE with enalapril consistently yielded needle‐like microcrystals that did not diffract X‐rays. To obtain the enalaprilat bound structure of nACE, we soaked enalapril into drops containing native crystals. Soaking significantly reduced the diffraction quality of the crystals and did not yield a bound structure. Subsequent observations of the native crystallisation drop used for soaking revealed the presence of new crystals. Diffraction data were collected on these crystals to obtain the nACE:enalaprilat structure.

**Fig. 2:**
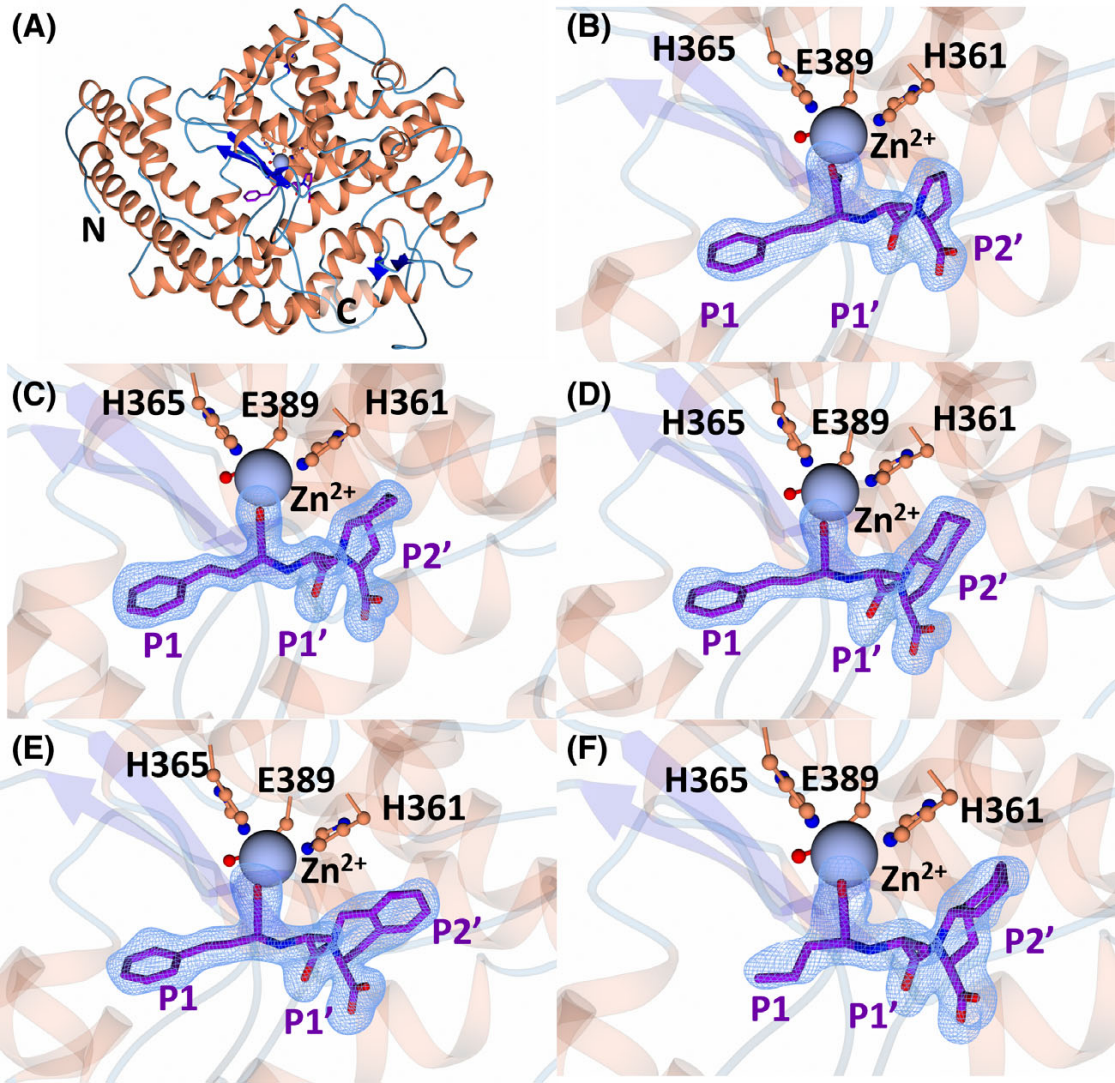
Crystal structures of nACE (N‐domain of human angiotensin I‐converting enzyme) in complex with enalaprilat, ramiprilat, trandolaprilat, quinaprilat, and perindoprilat. (A) Overall structure of nACE, as illustrated by nACE in complex with enalaprilat. α‐Helices are shown in orange, β‐sheets are shown in blue, loops are in light blue, inhibitors are in purple and zinc is in silver. The unbiased difference electron density map (Fo‐Fc, contoured at 3σ) calculated in the absence of modelled (B) enalaprilat, (C) ramiprilat, (D) trandolaprilat, (E) quinaprilat, and (F) perindoprilat are shown by the blue mesh. Figure was produced using ccp4mg.

**Fig. 3:**
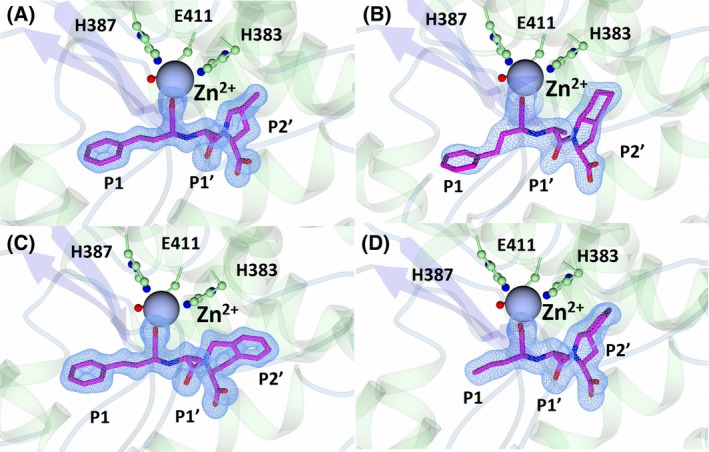
Crystal structures of cACE (C‐domain of human angiotensin I‐converting enzyme) in complex with ramiprilat, trandolaprilat, quinaprilat, and perindoprilat. α‐Helices are shown in green, β‐sheets are shown in blue, loops in light blue, inhibitors in magenta, and the zinc atom in grey. The unbiased difference electron density map (Fo‐Fc, contoured at 3σ) calculated in the absence of modelled (A) ramiprilat, (B) trandolaprilat, (C) quinaprilat, and (D) perindoprilat is shown by the blue mesh. Figure was produced using ccp4mg.

The inhibitor‐bound crystal structures of nACE contain two molecules in the asymmetric unit (ASU). Superimposition of chain A and chain B of all nACE crystal structures reported here indicated minimal variation between the two molecules of the ASU (RMSD for Cα atoms of 0.46–0.64 Å). The overall fold of both nACE and cACE in each structure was consistent with that which has been observed previously (Fig. 2A). This fold is comprised of a predominantly α‐helical secondary structure that makes up two distinct subdomains, subdomain 1 and subdomain 2, with an N‐terminal lid‐like region that has been shown to open and close [17, 34, 35, 36, 37]. Clear Fo‐Fc electron density for all the inhibitors (enalaprilat, ramiprilat, trandolaprilat, quinaprilat and perindoprilat) was observed at the active site of nACE (Fig. 2B–F) and cACE (Fig. 3A–D). Co‐crystallisation with the inhibitors was achieved using the prodrugs (enalapril, ramipril, quinapril, trandolapril, and perindopril), and thus, cleavage of the ethyl ester to the free‐acid form likely occurs within the crystallisation buffer. This was previously observed for cACE and AnCE (an ACE homologue from *Drosophila melanogaster*) in complex with enalaprilat [24, 38]. The X‐ray diffraction data collection and refinement statistics are shown in Table 2.

**Table 2:** X‐ray diffraction data collection and refinement statistics. The outer shell statistics are shown in parentheses. Average B‐factors, Ramachandran statistics, and clash scores were all calculated using molprobity. B‐factors for ions coordinating molecule A and molecule B of nACE are shown as A/B.

	nACE: enalaprilat	nACE: ramiprilat	nACE: quinaprilat	nACE: trandolaprilat	nACE: perindoprilat	cACE: ramiprilat	cACE: quinaprilat	cACE: trandolaprilat	cACE perindoprilat
Resolution (Å)	74.60–2.30	74.61–2.00	74.86–2.20	74.50–2.20	74.52–2.00	134.51–1.50	135.73–1.50	134.31–2.30	133.83–1.90
Space group	P1	P1	P1	P1	P1	P 2_1_ 2_1_ 2_1_	P 2_1_ 2_1_ 2_1_	P 2_1_ 2_1_ 2_1_	P 2_1_ 2_1_ 2_1_
Cell dimensions									
Lengths: *a*, *b*, *c* (Å)	72.3, 77.6, 81.0	73.3, 77.8, 83.4	72.84, 78.0, 81.7	73.2, 77.6, 82.5	73.3, 77.6, 83.2	56.7, 85.7, 134.5	59.7, 85.7, 135.7	57.0, 84.9, 134.3	56.5, 85.1, 133.8
Angles: α, β, γ (°)	88.7, 64.9, 75.1	88.4, 64.0, 74.5	88.5, 64.6, 74.7	88.5, 64.4, 74.8	88.6, 64.2, 74.8	90.0, 90.0, 90.0	90.0, 90.0, 90.0	90.0, 90.0, 90.0	90.0, 90.0, 90.0
Molecules per ASU	2	2	2	2	2	1	1	1	1
Completeness (%)	98.6 (97.7)	98.1 (97.1)	98.5 (97.7)	99.3 (98.8)	99.2 (98.6)	100 (100)	100 (100)	100 (100)	100 (100)
*R*_meas_	0.191 (1.478)	0.046 (0.180)	0.099 (0.489)	0.068 (0.241)	0.158 (1.229)	0.117 (2.206)	0.143 (2.222)	0.329 (1.686)	0.243 (2.940)
*R*_pim_	0.100 (0.784)	0.024 (0.094)	0.052 (0.256)	0.036 (0.129)	0.085 (0.696)	0.032 (0.603)	0.039 (0.605)	0.091 (0.475)	0.066 (0.792)
<*I*/σ*I*>	5.4 (1.0)	18.6 (6.6)	9.1 (2.7)	12.4 (5.2)	5.3 (1.1)	11.5 (1.1)	9.5 (1.1)	6.6 (1.7)	8.3 (1.1)
CC_1/2_	0.987 (0.412)	0.998 (0.976)	0.991 (0.870)	0.996 (0.959)	0.967 (0.453)	0.999 (0.516)	0.998 (0.519)	0.992 (0.745)	0.997 (0.450)
Multiplicity	3.6 (3.5)	3.5 (3.6)	3.5 (3.6)	3.4 (3.4)	3.4 (3.0)	13.1 (13.2)	13.2 (13.4)	13.0 (12.5)	13.2 (13.4)
*R*_work_/*R*_free_	0.18/0.22	0.18/0.22	0.17/0.21	0.17/0.21	0.20/0.23	0.17/0.20	0.14/0.20	0.19/0.26	0.17/0.22
RMSD bonds (Å)	0.011	0.012	0.008	0.012	0.009	0.014	0.014	0.009	0.014
RMSD angles (°)	2.057	1.909	1.673	1.941	1.863	2.22	2.066	1.791	2.34
Ramachandran angles									
Favoured (%)	97.41	97.66	97.83	97.66	97.90	98.59	98.38	96.83	97.88
Allowed (%)	2.59	1.92	2.00	2.09	1.76	1.23	1.44	3.17	1.94
Outliers (%)	0.00	0.42	0.17	0.25	0.34	0.18	0.18	0.00	0.18
Average *B*‐factors (Å^2^)									
Protein	40	33	29	32	35	24	24	34	32
Ligands	53	46	50	44	48	45	44	58	51
Water	32	32	31	31	34	34	38	30	36
Cl‐1 (mol A/B)	34/31	16/26	16/21	16/23	18/27	15	15	25	24
Cl‐2	NA	NA	NA	NA	NA	15	16	22	23
Zn (mol A/B)	27/31	16/21	16/19	16/18	18/23	14	14	25	22
Number of non‐hydrogen atoms									
Protein	9875	9973	9922	9954	9840	4816	4746	4683	4682
Ligands	321	335	369	340	252	140	154	144	137
Ions	5	7	6	7	4	3	4	3	3
Water	174	781	549	727	619	577	608	199	296
Clash score	4	4	3	5	3	3	2	3	3
PDB code	9QAV	9QAR	9QAT	9QAS	9QAU	9QAN	9QAP	9QAO	9QAQ

### Analysis of ligand binding interactions

The residues of nACE and cACE that interact with enalaprilat, ramiprilat, trandolaprilat, quinaprilat and perindoprilat are summarised in Table 3, and described by subsite in detail below.

**Table 3:** List of interacting residues at the S1, S1′ and S2′ subsites. Equivalent residues of nACE and cACE are aligned, and those that do not form an interaction are shown in red. Residues that form hydrogen bonds/electrostatic interactions are shown in bold, water‐mediated interactions in italics, and hydrophobic/stacking interactions are underlined. The number of hydrophobic contacts within 4 Å at the S2′ subsite is shown in brackets.

Inhibitor	nACE			cACE		
	S1	S1′	S2′	S1	S1′	S2′
Enalaprilat	Ser333 Phe490 Thr496	**Tyr501** ***Glu362*** *Ala334* **His331** **Ala332** Thr358 **His491**	***Lys489*** **Gln259** **Tyr498** *Asp255* His361 (2) Tyr501 (6) Phe435 Phe505	Ser355 Phe512 Val518	**Tyr523** ***Glu384*** *Ala356* **His353** **Ala354** Val380 **His513**	***Lys511*** **Gln281** **Tyr520** *Asn277* His383 (1) Tyr523 (5) Phe457 (1) Phe527
Ramiprilat	Ser333 Phe490 Thr496	**Tyr501** ***Glu362*** *Ala334* **His331** **Ala332** Thr358 **His491**	***Lys489*** **Gln259** **Tyr498** *Asp255* His361 (3) Tyr501 (4) Phe435 (1) Phe505 (1)	Ser355 Phe512 Val518	**Tyr523** ***Glu384*** *Ala356* **His353** **Ala354** Val380 **His513**	**Lys511** **Gln281** **Tyr520** *Asn277* His383 (4) Tyr523 (3) Phe457 (1) Phe527 (1)
Quinaprilat	Ser333 Phe490 Thr496	**Tyr501** ***Glu362*** *Ala334* **His331** **Ala332** Thr358 **His491**	***Lys489*** **Gln259** **Tyr498** *Asp255* His361 (2) Tyr501 (6) Phe435 (4) Phe505 (4)	Ser355 Phe512 Val518	**Tyr523** ***Glu384*** *Ala356* **His353** **Ala354** Val380 **His513**	**Lys511** **Gln281** **Tyr520** *Asn277* His383 (2) Tyr523 (5) Phe457 (2) Phe527
Trandolaprilat	Ser333 Phe490 Thr496	**Tyr501** ***Glu362*** *Ala334* **His331** **Ala332** Thr358 **His491**	***Lys489*** **Gln259** **Tyr498** *Asp255* His361 (4) Tyr501 (6) Phe435 (2) Phe505 (3)	Ser355 Phe512 Val518	**Tyr523** ***Glu384*** *Ala356* **His353** **Ala354** Val380 **His513**	**Lys511** **Gln281** **Tyr520** *Asn277* His383 (2) Tyr523 (2) Phe457 (1) Phe527 (1)
Perindoprilat	Ser333 Phe490 Thr496	**Tyr501** ***Glu362*** *Ala334* **His331** **Ala332** Thr358 **His491**	***Lys489*** **Gln259** **Tyr498** *Asp255* His361 Tyr501 (3) Phe435 Phe505 (2)	Ser355 Phe512 Val518	**Tyr523** ***Glu384*** *Ala356* **His353** **Ala354** Val380 **His513**	***Lys511*** **Gln281** **Tyr520** *Asn277* His383 (1) Tyr523 (2) Phe457 (1) Phe527 (1)

#### S1 subsite interactions

**Fig. 4:**
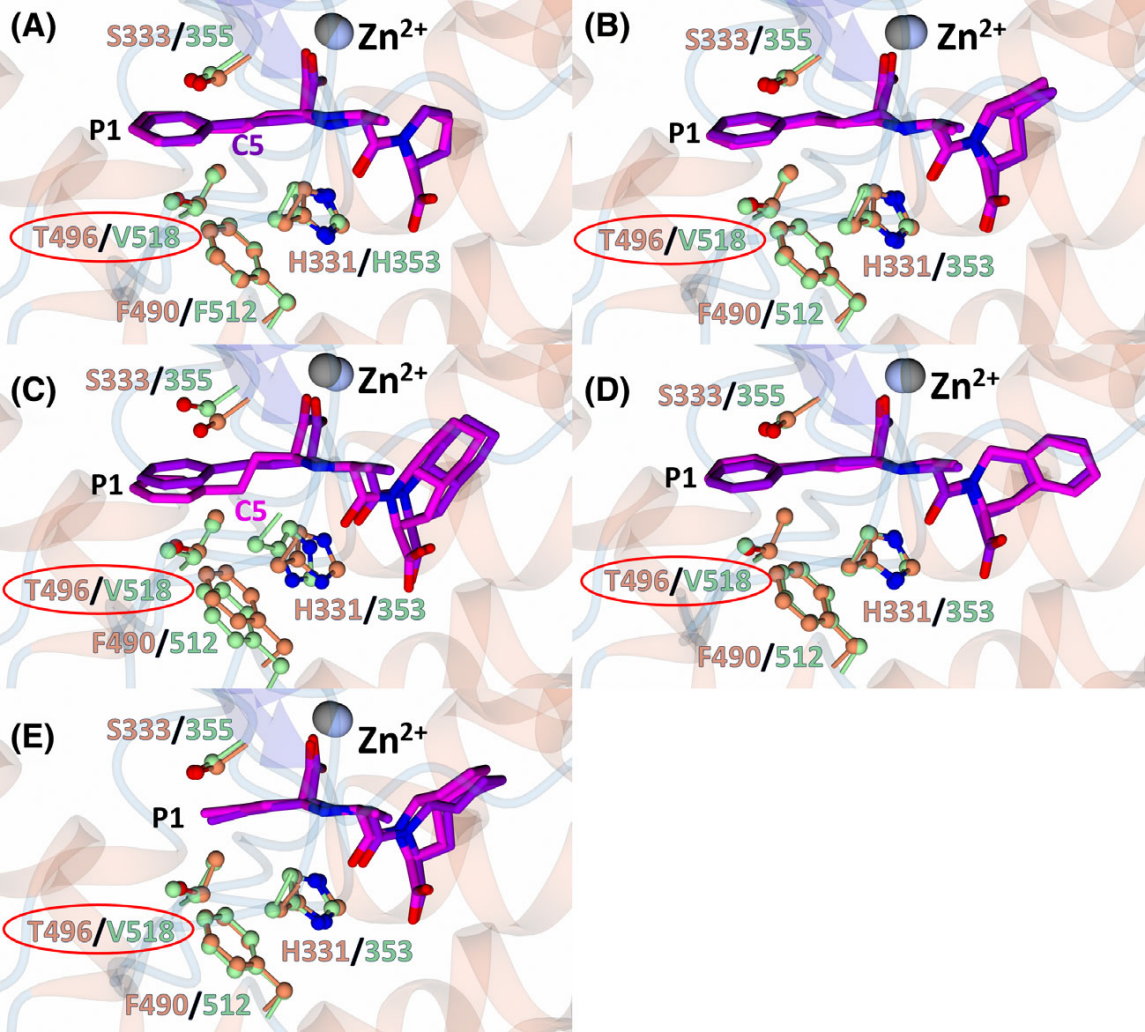
S1 subsite interactions of nACE (N‐domain of human angiotensin I‐converting enzyme) and cACE (C‐domain of human angiotensin I‐converting enzyme) in complex with enalaprilat, ramiprilat, trandolaprilat, quinaprilat, and perindoprilat. Superimposition of the nACE and cACE structures in complex with (A) enalaprilat, (B) ramiprilat, (C) trandolaprilat, (D) quinaprilat, and (E) perindoprilat. nACE residues are shown in orange and cACE in green. The inhibitors in complex with nACE are purple and in complex with cACE magenta. Residues that differ between nACE and cACE are shown by a red circle. The zinc atom in nACE and cACE is shown in silver and gray, respectively. Figure was produced using ccp4mg.

Enalaprilat, ramiprilat, trandolaprilat and quinaprilat all contain a phenyl‐propyl P1 moiety, whereas perindoprilat contains a propyl moiety. Both moieties occupy the hydrophobic S1 subsite formed by nACE‐Ser333 (cACE‐Ser355), nACE‐Phe490 (cACE‐Phe512) and nACE‐Thr496 (cACE‐Val518), except perindoprilat makes fewer hydrophobic interactions due to the less bulky propyl group (Fig. 4A–E). The P1 phenyl‐propyl moieties adopt a similar conformation in all structures, except for the nACE:enalaprilat (molecule B in the ASU) and cACE:trandolaprilat structures, where the C5 atom has shifted by ~ 1.5 Å due to rotation of the C3–C4 bond (Fig. 4A,C). This shift is towards the more hydrophobic environment formed by nACE‐Phe490 (cACE‐Phe512) and nACE‐His331 (cACE‐His353).

#### Catalytic zinc ion coordination and S1′ subsite interactions

**Fig. 5:**
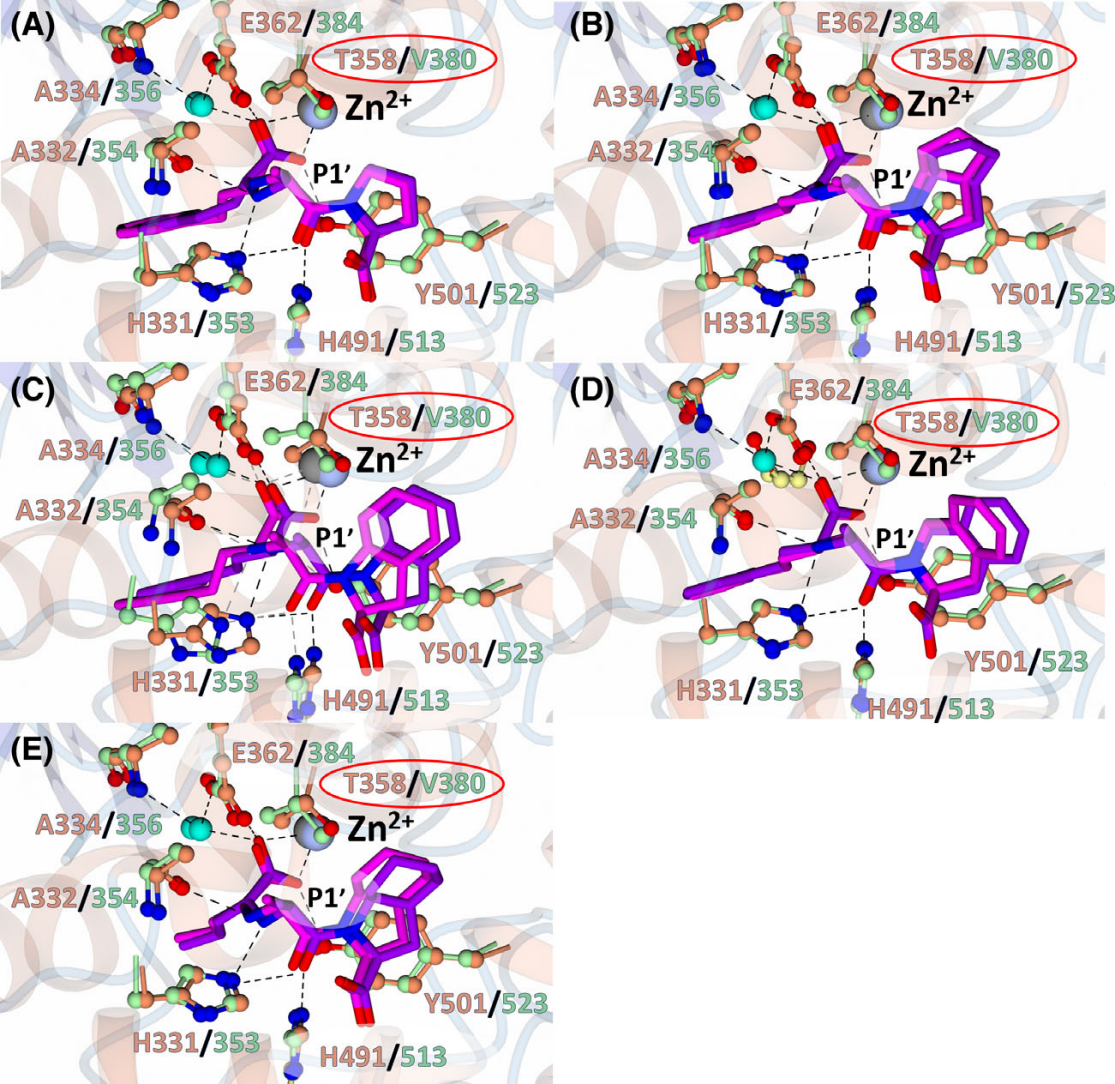
Zinc coordination and S1′ subsite interactions of nACE (N‐domain of human angiotensin I‐converting enzyme) and cACE (C‐domain of human angiotensin I‐converting enzyme) in complex with enalaprilat, ramiprilat, trandolaprilat, quinaprilat, and perindoprilat. Superimposition of the nACE and cACE structures in complex with (A) enalaprilat, (B) ramiprilat, (C) trandolaprilat, (D) quinaprilat, and (E) perindoprilat. nACE residues are shown in orange and cACE in green. The inhibitors in complex with nACE are purple and in complex with cACE magenta. Residues that differ between nACE and cACE are shown by a red circle. The zinc atom in nACE and cACE is shown in silver and gray, respectively. Water molecules are shown by cyan spheres. Figure was produced using ccp4mg.

Due to conservation of the carboxylate zinc‐coordinating group and P1′ moiety, the interactions observed for nACE and cACE in complex with enalaprilat, ramiprilat, trandolaprilat, quinaprilat and perindoprilat at the zinc and S1′ subsite are conserved. In all structures presented, the central carboxylate coordinates the zinc, in addition to forming a hydrogen bond with nACE‐Tyr501 (cACE‐Tyr523) and nACE‐Glu362 (cACE‐Glu384), and a solvent‐mediated interaction with nACE‐Ala334 (cACE‐Ala356) and nACE‐Glu362 (cACE‐Glu384). The P1′ alanine forms a hydrogen bond with nACE‐His331 (cACE‐His353) and nACE‐Ala332 (cACE‐Ala354) via its amine group, hydrophobic contacts with nACE‐Ala332 (cACE‐Ala354) and nACE‐Thr358 (cACE‐Val380) via the alanine side chain, and hydrogen bonds with nACE‐His331 (cACE‐His353) and nACE‐His491 (cACE‐His513) via its carbonyl group (Fig. 5A–E). These interactions likely favour binding to cACE due to substitution of nACE‐Thr358 for cACE‐Val380 providing a more hydrophobic environment for the P1′ alanine moiety.

#### S2′ subsite interactions

**Fig. 6:**
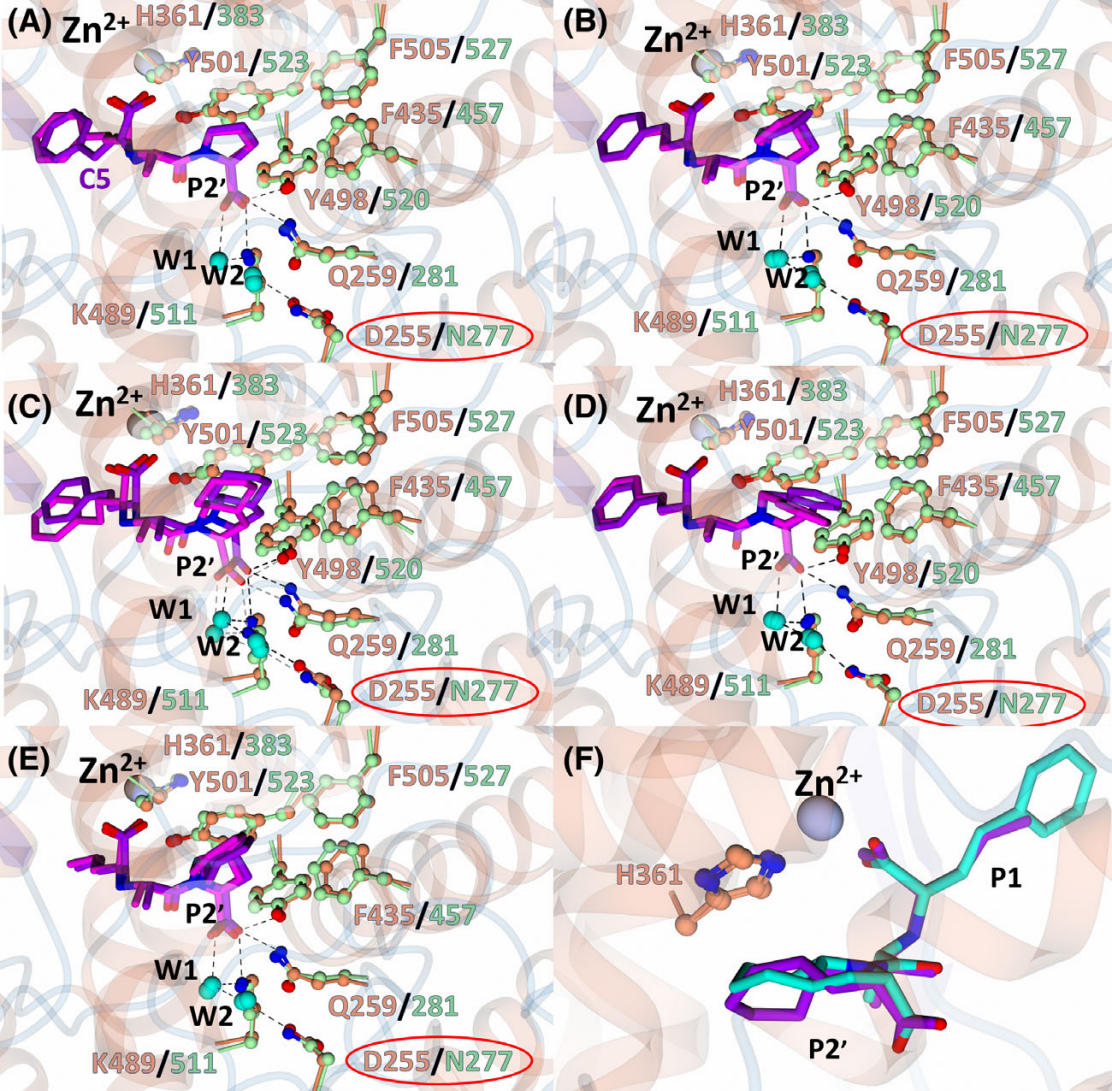
S2′ subsite interactions of nACE (N‐domain of human angiotensin I‐converting enzyme) and cACE (C‐domain of human angiotensin I‐converting enzyme) in complex with enalaprilat, ramiprilat, trandolaprilat, quinaprilat, and perindoprilat. Superimposition of the nACE and cACE structures in complex with (A) enalaprilat, (B) ramiprilat, (C) trandolaprilat, (D) quinaprilat, and (E) perindoprilat. nACE residues are shown in orange and cACE in green. The inhibitors in complex with nACE are shown in purple and in complex with cACE magenta. Residues that differ between nACE and cACE are shown by a red circle. The zinc atom in nACE and cACE is shown in silver and grey, respectively. Water molecules are shown by cyan spheres and labeled W1 and W2. (F) Comparison of different geometries of the P2′ moieties of perindoprilat (purple) and trandolaprilat (light blue). Only nACE:perindoprilat and nACE:trandolaprilat are shown for simplicity. Figure was produced using ccp4mg.

In each complex, the interactions with the terminal P2′ carboxylate group are conserved. These include a salt bridge with nACE‐Lys489 (cACE‐Lys511), hydrogen bonds with nACE‐Gln259 (cACE‐Gln281) and nACE‐Tyr498 (cACE‐Tyr520), and a two‐water‐mediated interaction with nACE‐Asp255 (cACE‐Asn277) (Fig. 6A–E). Enalaprilat contains the smallest P2′ moiety, a proline, which occupies the hydrophobic pocket formed by nACE‐Tyr501 (cACE‐Tyr523), nACE‐His361 (cACE‐His383), nACE‐Phe435 (cACE‐Phe457) and nACE‐Phe505 (cACE‐Phe527) (Fig. 6A). Ramiprilat, trandolaprilat, quinaprilat and perindoprilat contain larger P2′ moieties that also interact with nACE‐Tyr501 (cACE‐Tyr523), nACE‐His361 (cACE‐His383), nACE‐Phe435 (cACE‐Phe457) and nACE‐Phe505 (cACE‐Phe527) (Fig. 6B–E); however, they vary in orientation and size, which likely contributes to the differing *K*_i_ values (Table 1). If we consider hydrophobic contacts between carbon atoms by applying a distance cut‐off of 4 Å [39], an increase in hydrophobic contacts with the S2′ subsite (Table 3) does not appear to show a trend with binding strength (Table 1). Trandolaprilat and perindoprilat both contain an octahydroindole P2′ moiety; however, the stereochemistry of the bridgehead carbon atoms differs. In perindoprilat, the hydrogen atoms of the bridgehead atoms are *cis*, whereas in trandolaprilat, they are *trans* (Table 1). This alters the geometry of the ring and affects how it occupies the S2′ subsite (Fig. 6F). In perindoprilat, the octahydroindole ring is bent and parallel to nACE‐His361 (cACE‐His383), forming fewer hydrophobic contacts in both nACE and cACE compared with trandolaprilat (Table 3). However, the effect this has on binding strength cannot be directly determined due to the variable P1 moieties when comparing trandolaprilat and perindoprilat.

### Comparison of nACE and cACE structures with bound inhibitors

#### Enalaprilat

The interactions observed in the nACE:enalaprilat crystal structure are identical to those observed in the previously determined cACE crystal structure in complex with enalaprilat (cACE:enalaprilat, PDB 1UZE) [24], with minor differences in hydrophobic contacts. The P1 moiety in nACE:enalaprilat (Molecule B of the ASU) has rotated by ~ 137° (about the C3–C4 bond) and C5 has moved away from nACE‐Tyr501 (cACE‐Tyr523) at the S2′ subsite (Fig. 6A), compared to cACE:enalaprilat. This may be due to the flexibility of the P1 moiety, as it does not make any strong polar interactions. There is also a slight change in the position of nACE‐Phe435 (cACE‐Phe457) and nACE‐Phe505 (cACE‐Phe527) (Fig. 6A), where, relative to cACE, nACE‐Phe435 has rotated ~ 18° (about the Cβ–Cγ bond) and Phe505 ~ 6° (about the Cα–Cβ bond). This is a feature that is consistent across previously determined nACE structures when compared to cACE structures. These observations explain why enalaprilat displays no domain selectivity (3.6‐fold) (Table 1).

#### Ramiprilat

A comparison of nACE:ramiprilat to cACE:ramiprilat by superimposition revealed near perfect alignment of ramiprilat within the active site. The change in orientation of cACE‐Phe457 (nACE‐Phe435) and cACE‐Phe527 (nACE‐Phe505) only slightly alters the hydrophobic contacts with the P2′ moiety between nACE and cACE (Fig. 6B). Kinetic analysis shows that ramiprilat displays high inhibition but low domain selectivity (Table 1), with a cACE‐selectivity factor of 18.6. Therefore, the subtle differences at the S2′ subsite appear to have only minimal effects on ramiprilat binding.

#### Trandolaprilat

**Fig. 7:**
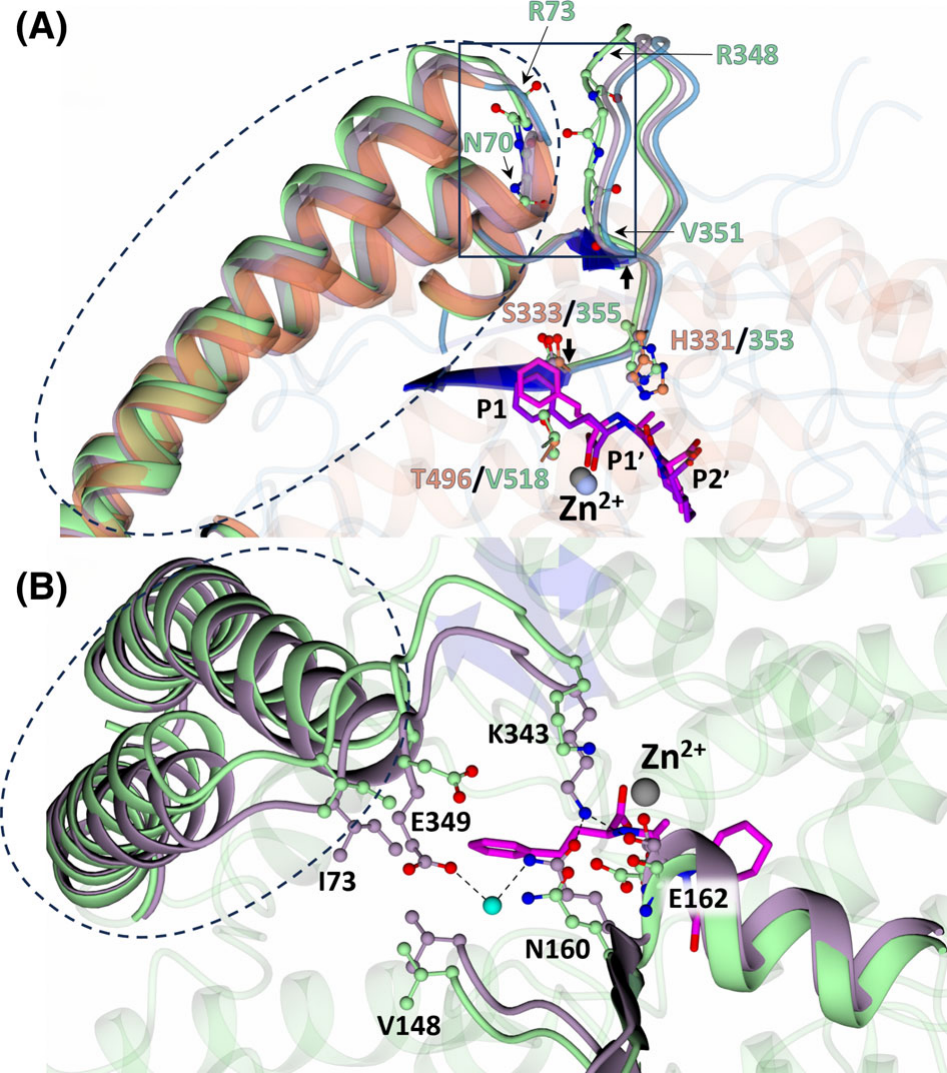
Conformational change associated with trandolaprilat binding in cACE (C‐domain of human angiotensin I‐converting enzyme). (A) Comparison of nACE:trandolaprilat, cACE:trandolaprilat, and native cACE at subdomain 1 reveals partial opening of the lid‐like region. Trandolaprilat in complex with nACE (N‐domain of human angiotensin I‐converting enzyme) is shown in purple, and cACE in magenta. The β‐stacking interaction between the lid‐like region and subdomain 1 is shown by the black square. Loop 341–355 is shown by the black arrows. (B) Comparison of interactions between subdomain 1 and 2 of cACE:trandolaprilat and native cACE. nACE:trandolaprilat is shown in orange (Panel A), cACE:trandolaprilat in green (Panels A and B), and native cACE in lilac (Panels A and B). The lid‐like region is shown by the dotted oval. Figure was produced using ccp4mg.

Superimposition of cACE:trandolaprilat with nACE:trandolaprilat revealed a shift in the relative position of trandolaprilat and the S1′ subsite residues cACE‐His353 (nACE‐His331), cACE‐Phe512 (nACE‐Phe490) and cACE‐His513 (nACE‐His491) (Figs 4C and 5C). The shift appears to be associated with the opening of the lid‐like region in cACE:trandolaprilat by ~ 7.4° (calculated using dyndom [40]) relative to nACE:trandolaprilat (Fig. 7), but does not alter the ligand binding interactions observed. The β‐stacking interactions between the lid‐like region and subdomain 1, comprised of residues 43–46/70–73 (nACE/cACE lid‐like region residues) and 327–329/348–350 (nACE/cACE subdomain 1 residues), are also retained upon the partial opening of cACE (Fig. 7A), a feature that is observed when comparing the closed nACE crystal structure with that of the ‘open’ nACE crystal structure [34]. This may explain the movement of cACE‐His353 upon the partial opening of the lid‐like region, as the cACE 341–355 loop (nACE 319–333 loop) must move to retain interactions between cACE residues 70–73 and 348–350 (Fig. 7A). When comparing cACE:trandolaprilat with the high‐resolution ‘closed’ structures of cACE in complex with fosinoprilat (cACE:fosinoprilat, PDB 7Z70) and the apo cACE structure (PDB code 2IUL), there appears to be a loss of interactions between residues cACE‐Lys343 and cACE‐Glu349 (water mediated) of subdomain 1 and residues cACE‐Gln160, cACE‐Glu162, and cACE‐Val148 of subdomain 2 in the cACE:trandolaprilat structure (Fig. 7B). The previously determined structure of cACE in complex with bradykinin potentiating peptide b (cACE:BPPb, PDB 4APJ) and sampatrilat (cACE:sampatrilat, PDB code 6F9T) also displayed movement within the lid‐like region (relative to apo cACE). In cACE:BPPb, this was associated with the rotation of cACE‐His383 away from the active site and displacement of the catalytic zinc upon BPPb binding, in addition to movement of cACE‐His353. In cACE:sampatrilat the lid‐like movement is also accompanied by movement of cACE‐His353, but to a further extent away from the active site [41]. Interestingly, of the complexes in the present study, cACE:trandolaprilat possesses the lowest *K*_i_ value (Table 1). Whether this is associated with the disruption of interactions between subdomains 1 and 2 requires further exploration; however, previous work conducted by our group has revealed the impact of distal residues on inhibitor binding [42].

#### Quinaprilat

**Fig. 8:**
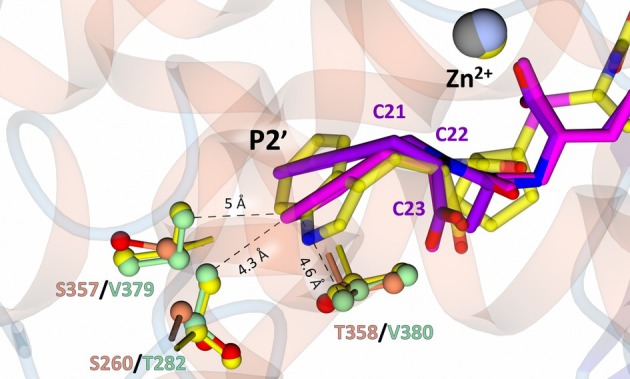
Comparison of quinaprilat binding in nACE (N‐domain of human angiotensin I‐converting enzyme) and cACE (C‐domain of human angiotensin I‐converting enzyme) with the cACE selective inhibitor RXPA380 at the S2′ subsite. Quinaprilat is shown in purple and magenta for nACE and cACE, respectively. RXPA380 is shown in yellow. Side chain carbon atoms for nACE:quinaprilat, cACE:quinaprilat, and cACE:RXPA380 are shown in orange, green, and yellow, respectively. All oxygen atoms are shown in red. Distances between quinaprilat in cACE and the side chains are shown by the dotted line. Zinc atoms are shown in silver, grey, and yellow for nACE:quinaprilat, cACE:quinaprilat, and cACE:RXPA380, respectively. Figure was produced using ccp4mg.

Quinaprilat displays the highest selectivity of the compounds presented here, with a cACE‐selectivity factor of 34.9. It contains an isoquinoline P2′ ring with a conjugated‐π system that limits the flexibility of the moiety. Superimposition of nACE:quinaprilat with cACE:quinaprilat shows that the P1 moiety aligns well between nACE and cACE (Fig. 4D). However, the P2′ moiety in cACE bends by ~ 7.65° (C21–C22–C23 angle), relative to nACE, towards the P2 carboxylate (Fig. 8). This positions the carbon atoms of the ring closer to the unique cACE residues, cACE‐Val379 (nACE‐Ser357), cACE‐Val380 (nACE‐Thr358) and cACE‐Thr282 (nACE‐Ser260). The high cACE‐selectivity of compound RXPA380 was determined to be, at least in part, due to van der Waal's contacts it may make with residues cACE‐Val379 (nACE‐Ser357) and cACE‐Val380 (nACE‐Thr358) [43]; it is possible that these residues also contribute to the cACE‐selectivity observed for quinaprilat. Carbon atoms of cACE quinaprilat are within ~ 4.3–5.0 Å of these side chains, making van der Waal's interactions likely.

#### Perindoprilat

Superimposition of nACE:perindoprilat with cACE:perindoprilat revealed near identical positioning of perindoprilat within the active site of nACE and cACE with a subtle displacement of the P2′ moiety (Fig. 6E). This is likely due to the shape of the S2′ subsite being altered slightly due to the minor differences in the positioning of residues nACE‐His361 (cACE‐His383), nACE‐Phe505 (cACE‐Phe527), and nACE‐Phe435 (cACE‐Phe457). The selectivity factor of perindoprilat is low (Table 1), and thus, these changes do not appear to contribute significantly to selectivity, given that all interactions are conserved.

### Effect of altering hydrophobic contacts at the S2′ subsite

The effect of differing hydrophobic contacts at the S2′ subsite was previously demonstrated using the phosphinic ACE inhibitor fosinoprilat [44]. The kinetic and structural characterisation of nACE and cACE in complex with fosinoprilat revealed a 27.4‐fold cACE selectivity, due to greater hydrophobic contacts with the P2′ moiety. Similar interactions at the S2′ subsite were observed for the highly cACE selective compound RXPA380 [43, 45]. In fosinoprilat, the selectivity was shown to be primarily influenced by amino acid differences at the S2′ subsite (nACE‐Thr358/cACE‐Val380, nACE‐Ser357/cACE‐Val379 and nACE‐Glu431/cACE‐Asp453). Despite being marginally cACE selective (Table 1), enalaprilat, trandolaprilat and quinaprilat make a greater number of hydrophobic contacts with nACE at the S2′ subsite (Table 3). This is in contrast to that which was proposed for fosinoprilat; however, the P2′ moieties of these inhibitors are not large enough to form interactions with the unique nACE/cACE residues. This is likely a contributing factor to the weak selectivity observed for these compounds. Interestingly, quinaprilat, which is the compound that is the most cACE selective out of those in the present study (Table 1), is positioned further into the S2′ subsite, towards the unique nACE/cACE residues, due to the presence of a piperidine ring in place of the pyrrole ring in enalaprilat, ramiprilat and trandolaprilat. The positioning of the hydrophobic P2′ moiety closer to the more polar S2′ subsite residues may contribute to destabilisation of quinaprilat in nACE. Furthermore, the P2′ moiety in quinaprilat is less flexible, compared to ramiprilat and trandolaprilat, which may also contribute to less favourable binding in nACE. This further illustrates that designing compounds to target the S2′–S3′ subsite is a promising strategy for the development of cACE selective inhibitors. Interestingly, variation of the P2′ moieties (relative to enalaprilat) appears to favour binding to cACE by a combination of increasing affinity for cACE (ramiprilat and trandolaprilat) and decreasing affinity for nACE (ramiprilat and quinaprilat). However, a comparison of the log*P* values, which quantifies the lipophilicity of drugs, for enalaprilat, ramiprilat, trandolaprilat and quinaprilat, with the Ki values reported here, indicates that increasing lipophilicity of the P2′ moiety does not directly result in an increase in affinity at the S2′ subsite (Table 1). Therefore, future drug development strategies would benefit from screening an extensive number of varying P2′‐P3′ moieties to determine functional groups that will improve cACE binding and/or reduce binding to nACE. Other clinically available ACE inhibitors, such as cilazapril, benazepril, moexipril, imidapril, delapril and spirapril, may offer further insight into the effects of altering the P2′ moiety on cACE‐selectivity due to their variable P2′ moieties (relative to enalaprilat).

## Conclusion

In the present study, we have reported high‐resolution crystal structures of nACE and cACE in complex with the clinically available ACE inhibitors enalaprilat, ramiprilat, trandolaprilat, quinaprilat and perindoprilat. Analysis of the binding and kinetics data illustrates minimal selectivity for cACE by all the inhibitors tested. Binding does not extend beyond the S1 or S2′ subsites (or deep into the S1′ subsite) to make use of the unique residues between nACE and cACE that have been shown (in some cases) to enhance inhibitor selectivity [43, 45, 46, 47, 48, 49, 50]. The only differences in residues that make up the S1 to S2′ subsites and interact with enalaprilat, ramiprilat, trandolaprilat, quinaprilat and perindoprilat are nACE‐Thr496 to cACE‐Val518 in the S1 subsite and nACE‐Thr358 to cACE‐Val380 in the S2′ subsite. These substitutions are likely to favour cACE binding due to enhanced hydrophobic contacts and may contribute to the subtle cACE selectivity observed. Although the domain selectivity for all inhibitors was shown to be poor to moderate, quinaprilat displays the highest selectivity amongst those reported here, and this is attributed to its orientation within the S2′ subsite, where it is positioned closer to the cACE‐specific residues (cACE‐Thr282, cACE‐Val379, and cACE‐Val380). However, the distances (4.3–5.0 Å) are not indicative of strong interactions. Furthermore, trandolaprilat was shown to have the highest affinity for both nACE and cACE, with a comparison of the nACE:trandolaprilat and native cACE structures to cACE:trandolaprilat revealing a partial opening of the lid‐like region. This opening was associated with disruption of interactions across the subdomains 1 and 2 interface in cACE. The role these interfacing residues have on inhibitor binding and substrate catalysis requires further detailed exploration.

Thus, while these clinical ACE inhibitors show moderate‐to‐poor domain selectivity, this comprehensive study of their interactions with cACE and nACE further indicates that targeting the S2′‐S3′ subsite is a promising strategy for the design of more selective cACE inhibitors.

## Materials and methods

### Reagents

For kinetics analysis, the ACE inhibitors (free‐acid form) were supplied by Cambridge Bioscience (Cambridge, UK). For crystallographic analysis, the ACE inhibitors were supplied by Merck (Gillingham, UK).

### Protein production

For kinetic analysis, a soluble human cACE construct from truncated testis ACE at Ser625 and lacking the first 36 heavily O‐glycosylated residues was used. For nACE, a soluble construct comprising the first 629 amino acids of human sACE (1–629) was used. Crystallisation studies employed minimally glycosylated and truncated nACE (N389) and cACE (g1,3) (Ser‐1 to Pro‐633). Proteins were expressed in cultured mammalian CHO‐K1 (CCL‐61™, obtained from ATCC (Manassas, VA, USA); RRID: CVCL_0213) cells. Soluble ACE was expressed in mycoplasma‐free cells and purified from the culture medium using lisinopril–sepharose affinity chromatography as previously described [50, 51].

### Inhibition assays

The IC_50_ values for the reversible, competitive inhibitors enalaprilat, ramiprilat, quinaprilat, trandolaprilat and perindoprilat with nACE and cACE were determined using a modified Z‐phenylalanyl‐histidyl‐leucine (Z‐FHL) assay (Bachem, Bubendorf, Switzerland) as previously described [32]. Briefly, inhibitors were dissolved in DMSO to stock concentrations of 10 mm (quinaprilat and trandolaprilat) and 50 mm (enalaprilat, ramiprilat and perindoprilat). Both nACE (20 nm) and cACE (9 nm) were incubated with a range of inhibitor concentrations (100 pm–10 μm) in phosphate buffer (0.1 m KPO_4_ buffer, pH 8.3 containing 0.3 m NaCl, 10 μm ZnSO_4_). The enzyme and inhibitor mixtures were then incubated with equal volumes of 1 mm Z‐FHL for 15 min. HL product formation results in fluorescence following derivitisation with *o*‐phthaldialdehyde, which was measured using a fluorescence spectrophotometer (Cary Eclipse; Varian‐ Santa Clara, CA, USA). IC_50_ values for nACE and cACE were determined from dose response curves plotted in prism, graphpad v.10 (GraphPad Software, Boston, MA, USA). *K*_i_ values were subsequently calculated by applying the Cheng–Prusoff equation (where [S] is the final substrate concentration of 0.5 mm Z‐FHL, and *K*_m_ is the substrate concentration (in the absence of inhibitor) at which the velocity of the reaction is half‐maximal) [51].

$$K_{i}=\frac{IC_{50}}{1+\frac{\left[S\right]}{K_{m}}}$$

cACE‐selectivity factors were calculated by *K*_i_(nACE)/*K*_i_(cACE).

### Crystallisation and structure determination

Prior to crystallisation trials, nACE and cACE were concentrated to 5 and 8 mg·mL^−1^, respectively. For all complexes, the prodrugs (enalapril, ramipril, trandolapril, quinapril and perindopril) were used for crystallisation trials. Ramipril and trandolapril were dissolved readily in DMSO, and enalapril, perindopril, and quinapril in water. For each complex, 3 μL of protein (nACE or cACE) was incubated with 0.75 μL ([10 mm] stock) of inhibitor (enalapril, ramipril, trandolapril, quinapril, or perindopril) for at least 1 h (for nACE this was performed at room temperature, and for cACE this was performed on ice). nACE crystals grew in 30% PEG 550, MME/PEG 2000, 0.1 m Tris/bicine pH 8.5 and cACE in 0.1 m MIB pH 4.0, 5% glycerol, 15% PEG 3350 at 16 °C. The nACE:enalapril crystals grown by cocrystallisation yielded a small shower of needles that did not diffract even with the use of the microfocus beamline on I04 (Diamond Light Source, Abingdon, UK). Attempts were made to soak enalapril into native crystals directly in the drop. Soaking significantly decreased the diffraction quality of native crystals and resulted in structures without enalaprilat bound. Soaking was performed by the addition of 1 μL enalapril ([20 mm] stock) inhibitor directly into the drop, which contained native crystals, and these drops were returned to the incubator. Subsequent inspection of these drops a month later indicated the presence of newly grown crystals, distinctly larger than the co‐crystals grown previously. These crystals diffracted, and the structure of nACE:enalapril could be determined. Crystals were mounted onto a cryoloop and cryocooled in liquid nitrogen for data collection at 100 K on beamline I04 (Diamond Light Source). A total of 3600 images were collected per dataset at 0.1° of oscillation. Exposure time per image was adjusted to ensure the dose did not exceed 14 MGy (3.00–13.50 MGy used depending on dataset) to limit radiation damage. Data were indexed and integrated in dials [52], and subsequently processed using the ccp4i2 suite for data reduction, scaling and merging in aimless [53], molecular replacement using phaser [54], refinement using refmac [55], and validation using molprobity [56]. Figures were produced in ccp4mg [57]. The tilt angle of cACE:trandolaprilat, relative to nACE:trandolaprilat, was calculated using dyndom [40].

## Conflict of interest

The authors declare no conflict of interest.

## Author contributions

KSG wrote the manuscript, performed crystallisation, X‐ray diffraction data collection, processed the data, analysed the data and edited the manuscript. VR performed the expression and purification of nACE and cACE and the kinetic characterisation of inhibitor binding. EDS supervised the kinetic study, analysed the data and edited the manuscript. KRA supervised the crystallographic study, analysed the data and edited the manuscript. All authors reviewed the manuscript.

## Data Availability

The structure factors and coordinates of crystal structures nACE:enalaprilat (9QAV), nACE:ramiprilat (9QAR), nACE:trandolaprilat (9QAS), nACE:quinaprilat (9QAT), nACE:perindoprilat (9QAU), cACE:ramiprilat (9QAN), cACE:trandolaprilat (9QAO), cACE:quinaprilat (9QAP) and cACE:perindoprilat (9QAQ) have been deposited to the protein data bank (PDB) and their corresponding PDB accession codes are shown in brackets.
